# 1328. Paenibacillosis: An Emerging Cause of Neonatal Sepsis and Postinfectious Hydrocephalus

**DOI:** 10.1093/ofid/ofac492.1158

**Published:** 2022-12-15

**Authors:** Jessica E Ericson, Kathy Burgoine, Christine Hehnly, Elias Kumbakumba, Moses Ochora, Francis Bajunirwe, Joel Bazira, Claudio Fronterre, Cornelia Hagmann, Abhaya Kulkarni, M Senthil Kumar, Joshua Magombe, Edith Mbabazi-Kabachelor, Sarah Morton, Mercedeh Movassagh, John Mugamba, Ronald Mulondo, Abraham Muwanguzi, Davis Natukwatsa, Brian Nsubuga Kaaya, Peter Olupot-Olupot, Justin Onen, Kathryn Sheldon, Jasmine Smith, Paddy Ssentongo, Peter Ssenyonga, Benjamin Warf, Emmanuel Wegoye, Lijun Zhang, James Broach, Julius Kiwanuka, Joseph Paulson, Steven Schiff

**Affiliations:** Penn State College of Medicine, Hershey, Pennsylvania; Mbale Regional Referral Hospital, Mbale, Uganda, Mbale, Mbale, Uganda; Penn State College of Medicine, Hershey, Pennsylvania; Mbarara University of Science and Technology, Mbarara, Mbarara, Uganda; Mbarara University of Science and Technology, Mbarara, Mbarara, Uganda; Mbarara University of Science and Technology, Mbarara, Mbarara, Uganda; Mbarara University of Science and Technology, Mbarara, Mbarara, Uganda; Lancaster University, London, England, United Kingdom; University of Zurich, Zurich, Zurich, Switzerland; University of Toronto, Toronto, Ontario, Canada; Harvard T.H. Chan School of Public Health and Dana Farber Cancer Institute, Boston, Massachusetts; CURE Children's Hospital of Uganda, Mbale, Mbale, Uganda; CURE Children's Hospital of Uganda, Mbale, Mbale, Uganda; Harvard Medical School, Boston, Massachusetts; Harvard T.H. Chan School of Public Health and Dana Farber Cancer Institute, Boston, Massachusetts; CURE Children's Hospital of Uganda, Mbale, Mbale, Uganda; CURE Children's Hospital of Uganda, Mbale, Mbale, Uganda; National Planning Authority, Kampala, Kampala, Uganda; CURE Children's Hospital of Uganda, Mbale, Mbale, Uganda; CURE Children's Hospital of Uganda, Mbale, Mbale, Uganda; Mbale Regional Referral Hospital; Busitema University, Mbale, Mbale, Uganda; CURE Children's Hospital of Uganda, Mbale, Mbale, Uganda; Penn State College of Medicine, Hershey, Pennsylvania; Penn State College of Medicine, Hershey, Pennsylvania; Penn State College of Medicine, Hershey, Pennsylvania; CURE Children's Hospital of Uganda, Mbale, Mbale, Uganda; Harvard Medical School, Boston, Massachusetts; CURE Children's Hospital of Uganda, Mbale, Mbale, Uganda; Penn State College of Medicine, Hershey, Pennsylvania; Penn State College of Medicine, Hershey, Pennsylvania; Mbarara University of Science and Technology, Mbarara, Mbarara, Uganda; Genentech Inc, San Francisco, California; Penn State College of Medicine, Hershey, Pennsylvania

## Abstract

**Background:**

The etiology of neonatal sepsis is often not identified. Molecular methods can identify pathogens that culture-based methods miss. Most cases of neonatal sepsis globally are treated empirically per WHO guidelines with intravenous ampicillin and gentamicin, which may not be the best regimen for all pathogens.

**Methods:**

We prospectively enrolled 800 neonates presenting with signs of sepsis to two Ugandan hospitals. Blood and cerebrospinal fluid were subjected to 16S rRNA sequencing, which identified *Paenibacillus thiaminolyticus* in 33/800 (4%) neonates. We confirmed the presence of *P. thiaminolyticus* by quantitative polymerase chain reaction (PCR). We describe neonatal and birth characteristics, presenting signs, and 12-month developmental outcomes for neonates with paenibacillosis. We performed antibiotic susceptibility testing and genomic analyses on three clinical isolates successfully grown in the laboratory.

**Results:**

Neonates presented at a median age of 3 (1, 7) days. Fever (86%), irritability (78%) and seizures (52%) were common presenting signs (Figure). Most neonates were born vaginally (73%) at a medical facility (79%). Twelve (36%) had an adverse outcome: 5 (15%) neonates died; 4 (14%) survivors developed postinfectious hydrocephalus and three (9%) additional survivors had neurodevelopmental impairment.

All three isolates were resistant to vancomycin, two were resistant to penicillin and ampicillin and one was unlikely to be sensitive to ceftriaxone; all were susceptible to gentamicin and meropenem. The genomes of all three strains contained multiple beta-lactamase genes and a cluster of genes that encodes a type IV pilus.

Clinical signs at presentation for neonates with good and poor outcomes followng paenibacillosis

**Figure:**
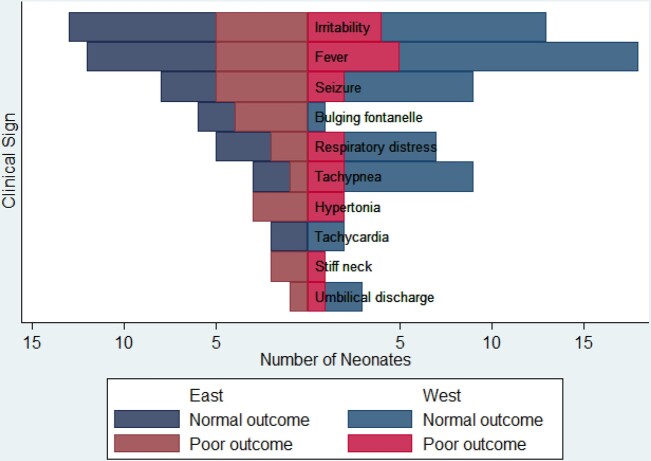

**Conclusion:**

Molecular methods such as 16S rRNA sequencing and PCR can be used to improve the identification of pathogens causing neonatal sepsis. Paenibacillosis is an important emerging cause of neonatal sepsis in Uganda and is likely an underrecognized cause of postinfectious hydrocephalus in the region and possibly elsewhere. Antibiotics commonly used for neonatal sepsis may be inadequate for the treatment of paenibacillosis. Additional studies to understand the pathophysiology and optimal treatment of this novel infection are urgently needed to prevent neonatal mortality and morbidity including postinfectious hydrocephalus.

**Disclosures:**

**Jessica E. Ericson, MD, MPH**, Abbvie: Advisor/Consultant **Abhaya Kulkarni, MD, MSc, PhD**, Medtronic: Advisor/Consultant.

